# An Information Theory Approach to Aesthetic Assessment of Visual Patterns

**DOI:** 10.3390/e23020153

**Published:** 2021-01-27

**Authors:** Abdullah Khalili, Hamid Bouchachia

**Affiliations:** Department of Computing and Informatics, Bournemouth University, Poole BH12 5BB, UK; abouchachia@bournemouth.ac.uk

**Keywords:** image aesthetic assessment, human–computer interaction, computer vision, evolutionary art, information theory

## Abstract

The question of beauty has inspired philosophers and scientists for centuries. Today, the study of aesthetics is an active research topic in fields as diverse as computer science, neuroscience, and psychology. Measuring the aesthetic appeal of images is beneficial for many applications. In this paper, we will study the aesthetic assessment of simple visual patterns. The proposed approach suggests that aesthetically appealing patterns are more likely to deliver a higher amount of information over multiple levels in comparison with less aesthetically appealing patterns when the same amount of energy is used. The proposed approach is evaluated using two datasets; the results show that the proposed approach is more accurate in classifying aesthetically appealing patterns compared to some related approaches that use different complexity measures.

## 1. Introduction

The study of aesthetics started with the work of ancient Greek, and today it is an active research topic in fields as diverse as neuroscience [1], psychology [2], and computer science. Baumgarten [3] suggested that aesthetic appreciation is the result of objective reasoning. Hume [4] took the opposing view that aesthetic appreciation is due to induced feelings. Kant argued that there is a universality aspect to aesthetic [5]. Shelley et al. [6] studied the influence of subjective versus objective factors in aesthetic appreciation. Recent studies on empirical aesthetics [7] show that there is a general agreement on what is considered beautiful and what is not, despite the subjectivity of aesthetic appeal. Measuring the aesthetic appeal of images is beneficial for many applications, such as recommendation and retrieval in multimedia systems. It also plays a key role in enhancing human–computer interaction by improving the attention, engagement, and the overall user experience. The development of a model of aesthetic judgment is also a major challenge in evolutionary art [8,9], where only images with high aesthetic quality should be generated. Automating the aesthetic assessment is still an open problem, and the development of models of aesthetic judgment is the main challenge. In this paper, a novel approach to classifying aesthetically appealing images will be presented. The main contribution of this paper is showing that aesthetically appealing patterns are more likely to deliver a higher amount of information over multiple levels in comparison with less aesthetically appealing patterns when the same amount of energy is used. The proposed approach is evaluated using two datasets.

## 2. Related Work

Datta et al. [10] extracted 56 visual features from an image and used them to train a statistical model to classify the images as “beautiful” or “ugly”. Some examples of the used features include: mean pixel intensity, relative color frequencies, mean pixel hue, and mean pixel saturation. They also used photographic rules of thumb such as the rule-of-thirds. Other features related to aspect ratio, texture, and low depth-of-field were also used. Ke et al. [11] used features that describe the spatial distribution of color, edges, brightness, and blur. Aydin et al. [12] computed perceptually calibrated ratings for a set of meaningful and fundamental aesthetic attributes such as depth, sharpness, tone and clarity, which together form an “aesthetic signature” of the image. Other works have also investigated the role of photographic composition [13,14,15,16], colour compatibility [17,18,19], and the use of other features such as object types in the scene [20]. Recently, convolutional neural networks (CNNs), which can automatically learn the aesthetic features, have been applied to the aesthetic quality assessment problem [21,22,23,24]; promising results were reported.

This research is more related to the information-theory-based approaches. Birkhoff [25] proposed an aesthetic measure, where the measure of aesthetic quality is in a direct relation to the degree of order O, and in a reverse relation to the complexity C, M = O/C. Eysenck [26,27,28] conducted a series of experiments on Birkhoff’s model; he argued that the aesthetic measure has to be in direct relation to the complexity rather than an inverse relation M = O × C. Javid et al. [29] conducted a survey on the use of entropy to quantify order and complexity; they also proposed a computational measure of complexity. Their measure is based on the information gain from specifying the spatial distribution of pixels and their uniformity and non-uniformity. Franke [30] proposed a model based on psychological experiments, which showed that working memory cannot take in more than 16 bits/s of visual information. He argued that artists should provide an information flow of about 16 bits/s for their works to be perceived as aesthetically appealing and harmonious; see [31] for more recent developments. Al-Rifaie et al. [32] proposed a nature-inspired, swarm intelligence technique to quantify symmetrical complexities in visual patterns. The technique is then used to investigate aesthetically appealing patterns. Javid et al. [33] investigated the use of Kolmogorov complexity and mean information gain to distinguish 2D patterns. The measures were able to distinguish between random patterns and non-random patterns. Datasets such as [34,35,36,37] are collected from communities where images are uploaded and scored in response to photographic challenges. The main limitation of these datasets is that the images are very rich, diverse, and highly subjective, which will make the aesthetic assessment process very complicated. Therefore, the datasets in [38] and [39] will be used in this paper to test the proposed approach. Using simple visual patterns in these two datasets was necessary to simplify the process and filter out unnecessary information as much as possible. The second reason for using these two datasets was to reduce the subjectivity of the assessment process as much as possible by using very simple patterns instead of using real-world images that have a more subjective nature, such as other datasets [34,35,36,37].

## 3. Proposed Approach

**Figure 1:**
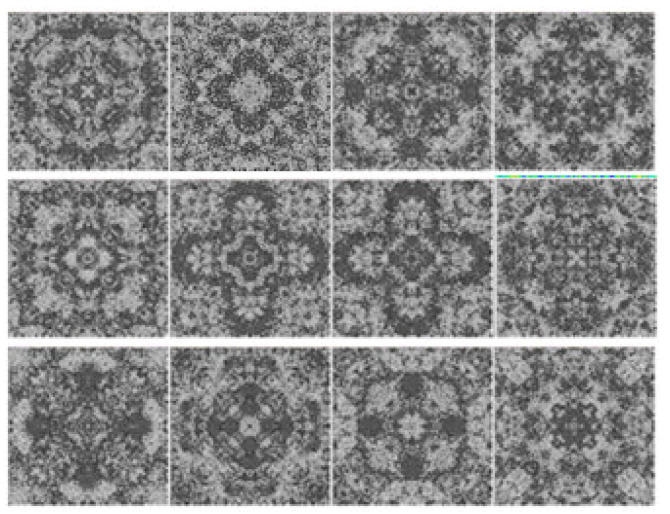
Images in the first group.

**Figure 2:**
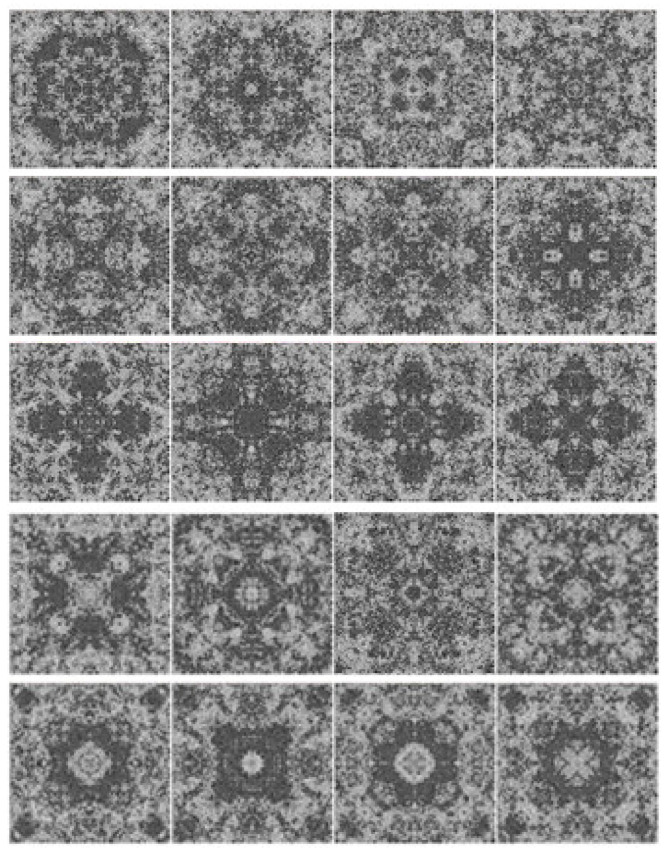
Images in the second group.

In this section, simple visual patterns will be studied. The images of the dataset in [38], and the images of the dataset in [39] will be used, the dataset in [38] contains two groups of images: the first one is “more aesthetically appealing” images (Figure 1), and the second one is “less aesthetically appealing” images (Figure 2). These two groups are rated by ten persons. The ten persons were asked to give a binary classification of whether each pattern is beautiful or not. If the score (the number of persons who selected the pattern as beautiful) is higher than the average score, then the pattern belongs to the first group, otherwise it belongs to the second group. The dataset contains simple visual patterns generated by the same physical process. The propagation of waves inside geometrical structures could produce very interesting interference patterns, particularly inside symmetrical shapes. The resulted pattern represents the wave interference pattern inside a closed box. Three waves were initiated at the center of the box at different time instances. The first wave was initiated when the value of the counter was 1, the second wave was initiated when the value of the counter was 5000, and the third wave was initiated when the value of the counter was 10,000. The size of the images is 116 × 116 pixels. The images are grayscale images with 256 possible values.

To analyze the images of Figure 1 and Figure 2, if we start from the center of the image to the boundary, we notice that the number of transitions between lighter and darker values is larger for images in Figure 1; furthermore, the intensity of the transitions is higher. This will result in increasing the high-energy part of the distribution of the gradient of the image. Moreover, we notice that the high-energy part of the distributions of the images of Figure 1 is larger than the high-energy part of the distributions of the images in Figure 2 when both have the same amount of energy, and since the largest part of the distribution is located in the low-energy region, this means that increasing the high-energy part of the distribution will increase the entropy.

The basic idea of the proposed approach is that aesthetically appealing patterns have a balance between randomness and regularity, and aesthetically appealing patterns are those which are closer to this optimal point. The entropy and energy will be used as measures of this balance. The resulted distribution of this optimization process can be uniquely identified by maximizing the entropy, given that the energy levels are constant, and the total energy is constant. 

The main difference of the proposed approach in comparison with existing approaches to aesthetic assessment of visual patterns is the use of a statistical mechanics formulation. The main reason for using this formulation is that it provides a link between the energy and the entropy, which was a crucial link to constrain the complexity of the pattern by the energy, and hence achieve a balance between randomness and regularity; this balance was also suggested by many researchers [40,41,42,43]. The approach does not assume any link to statistical mechanics, it only uses the same mathematical formulation.

**Figure 3:**
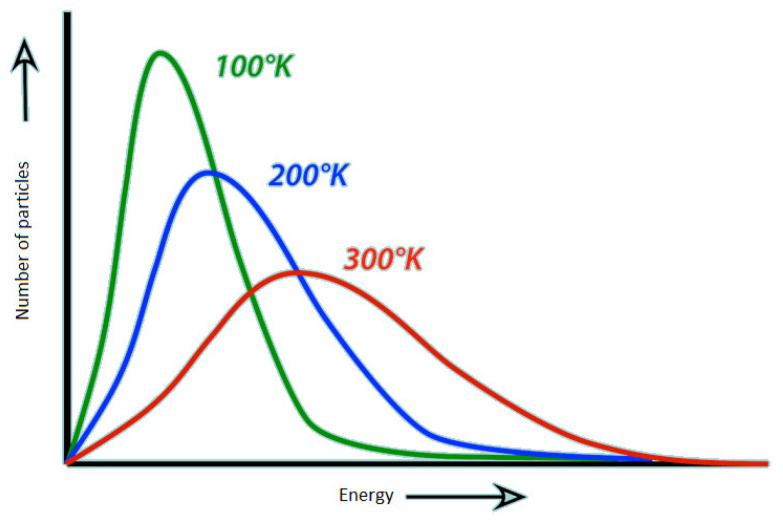
The Maxwell–Boltzmann distribution for different temperature values.

**Figure 4:**
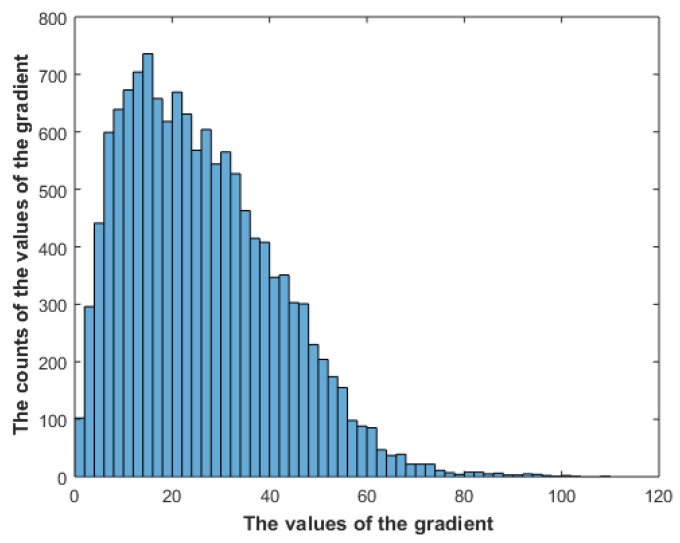
The distribution of the gradient of one image in the dataset.

Figure 3 shows the Maxwell–Boltzmann distribution, Figure 4 shows the distribution of the gradient of one image in the dataset; the same distribution has shown up for all the images in the dataset. We can observe the similarity between the resulting distribution and the Maxwell–Boltzmann distribution. Furthermore, using the above analysis, our problem now is exactly the same problem that Boltzmann [44] solved to derive the distribution of the energies of gas particles at equilibrium. Boltzmann argued that the Maxwell-Boltzmann distribution [45,46] is the most probable distribution and it will arise by maximizing the multiplicity (which is the number of ways the particles can be arranged); assuming that the number of particles is constant, as described by (1), the energy levels that the particles can take are constant, as described by (2), and the total energy is constant, as described by (3). The multiplicity is given by (4), and the entropy is given by (5)

(1) $$\underset{i}{\sum }n_{i}=Constant$$

(2) $$\varepsilon_{1},\;\varepsilon_{2},\;\ldots ,\;\varepsilon_{N}\;\;are\;constant$$

(3) $$\mathrm{Energy}\;=\underset{i}{\sum }n_{i}\varepsilon_{i}=Constant$$

(4) $$Ω=\;\frac{N!}{n_{1}!n_{2}!\ldots .n_{n}!}$$

(5) Entropy =log(Ω)

where *N* is the total number of particles, $n_{i}$ is the number of particles at the $\varepsilon_{i}$ energy level. Maximizing the entropy is equivalent to maximizing the multiplicity. By taking ln (Ω), we get

(6) $$\ln\left(Ω\right)\;=\ln\left(N!\right)-\underset{i}{\sum }\ln(n_{i}!)$$

Using Stirling approximation, Equation (6) can be rewritten as follows

(7) $$\ln\left(Ω\right)=N\ln\left(N\right)-N-\underset{i}{\sum }[n_{i}\ln(n_{i})-\;n_{i}]$$

The Maxwell–Boltzmann distribution gives the number of particles at each energy level. Using the Lagrange multiplier method to maximize the entropy using the constraints in (1)–(3), we get

(8) $$n_{i}=\;e^{-\alpha -\beta \varepsilon_{i}}$$

where α,β are the Lagrange multipliers. The distribution in 3D and 2D spaces can be written in the form given by (9) and (10), respectively,

(9) $$f\left(v\right)={\left(\frac{m}{2\pi kT}\right)}^{\frac{3}{2}}4\pi v^{2}\;e^{-\;\frac{mv^{2}}{2kT}}$$

(10) $$f\left(v\right)=\left(\frac{m}{2\pi kT}\right)2\pi v\;e^{-\;\frac{mv^{2}}{2kT}}$$

where v is the speed of the particle, m is the mass of the particle, T is the temperature and k is Boltzmann constant. The distribution is shown in Figure 3.

Similarly, for images, the energy levels $\varepsilon_{1},\;\varepsilon_{2},\;\ldots ,\;\varepsilon_{n}$ are the values which the pixels can take; for grayscale images, the values are 0, 1, 2, …, 255. The energy levels must be constant, as described in (11); $n_{i}$ is the number of pixels at the energy level $\;\varepsilon_{i}$, the total number of pixels should also be constant, as described in (12). Finally, the total energy, which is given by (13), must also be constant.

(11) $$\varepsilon_{1},\;\varepsilon_{2},\;\ldots ,\;\varepsilon_{n}\;are\;constant$$

(12) $$\underset{i}{\sum }n_{i}=Constant$$

(13) $$\mathrm{Energy}\;=\underset{i}{\sum }n_{i}\varepsilon_{i}=Constant$$

**Figure 5:**
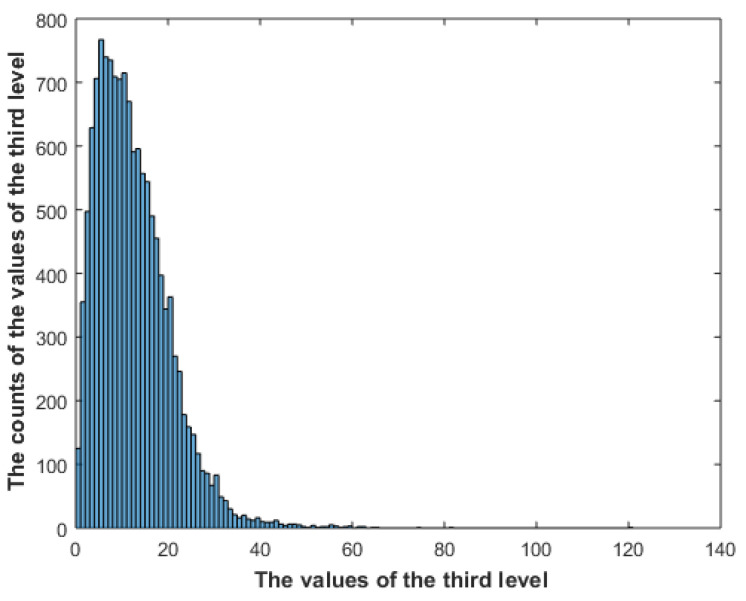
The distribution of the gradient of the gradient of one image in the dataset.

The constraints given in (11)–(13) are exactly the same constraints used by Boltzmann to derive the Maxwell–Boltzmann distribution, and by maximizing the entropy, the same distribution as given by (8)–(10) will arise. Maximizing the entropy will result in a flat distribution; however, the constant energy constraint will produce a balance between order and randomness. Maximizing the entropy using constant energy can then be seen as delivering the highest possible amount of information using the same amount of energy. Figure 4 shows the distribution of the gradient of an image in the dataset. The Matlab gradient function is used to calculate the gradient, and then the resulting values are converted to polar format. Figure 5 shows the distribution of the gradient of the gradient of the same image.

**Figure 6:**
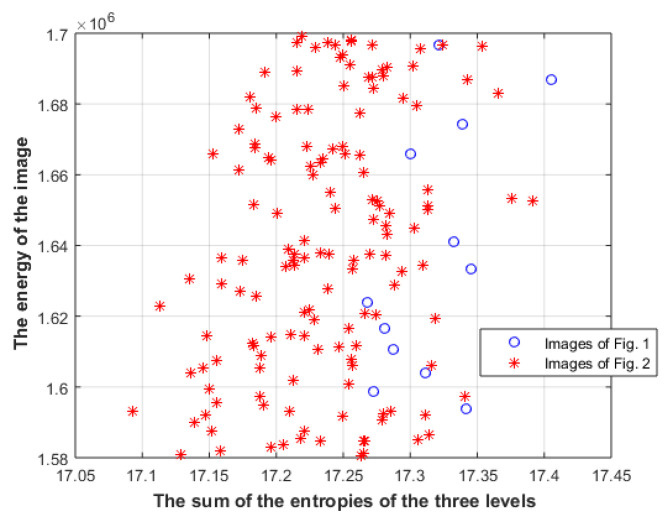
The M values of images in Figure 1 and Figure 2.

The same distribution has appeared for all the gradient of the images, and the gradient of the gradient of the images, which may suggest that the same law must be satisfied at each level. The multiple-levels approach will be used to cope with energy and entropy limitation in representing the spatial arrangement of the pattern. Due to the complexity of the structure of the visual patterns, the gradient over multiple levels will be used to represent the spatial arrangement of the visual patterns, where the first level represents the image, the second level represents the gradient of the image, and the third level represents the gradient of the gradient of the image. The measures of aesthetic quality M propose that the sum of the entropies of the three levels should be maximum when the energies of the three levels are the same. The measure is given by (14)

(14) $$M=\underset{i=1,3}{\sum }\mathrm{Entropy}\left(\mathrm{Li}\right)$$

where L_1_ is the image, L_2_ is the gradient of the image, and L_3_ is the gradient of the gradient of the image. Entropy is Shannon entropy (using Stirling approximation, Shannon entropy can be used instead of Boltzmann entropy), and the energies of the three levels must be the same. Figure 6 shows the M values of images in Figure 1 and Figure 2, along with other images in the same category. 

**Figure 7:**
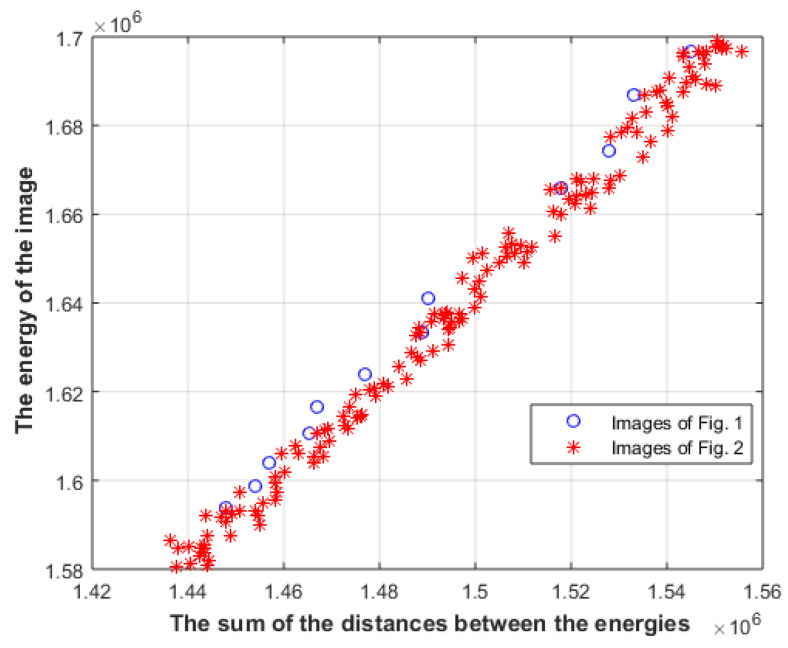
The sum of the distances between the energies of different levels for images in Figure 1 and Figure 2.

However, comparing images that have the same energy at each level is rather limited; furthermore, the above analysis does not say anything about the relation between the energies of different levels. Figure 7 shows the sum of the distances between the energies of different levels for images in Figure 1 and Figure 2, along with other images in the same category.

**Figure 8:**
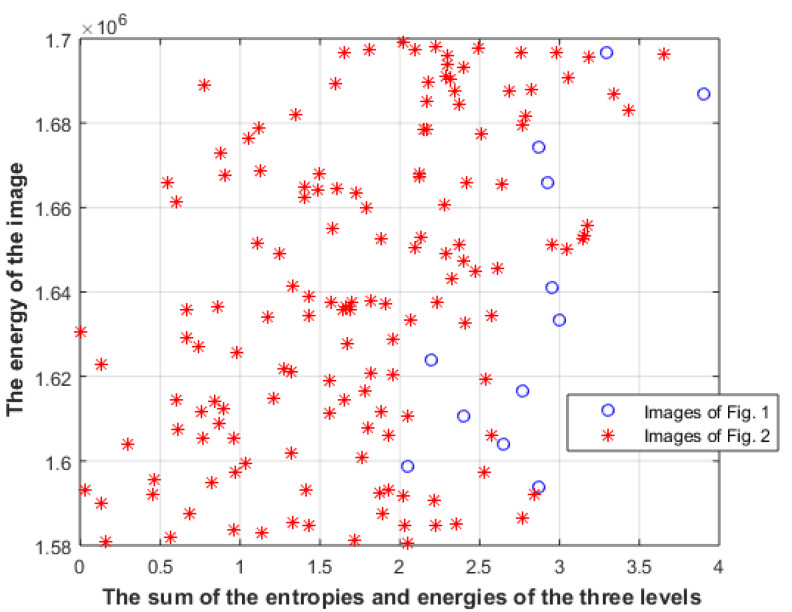
The sum of the entropies and energies of the three levels of images in Figure 1 and Figure 2.

The blue circles represent the images of Figure 1, and the red stars represent the images of Figure 2, along with other images in the same category. The distances of aesthetically appealing images are different from the distances of the less aesthetically appealing images. To relax the above constraint, and to be able to compare images that have the same first-level energy only, the aesthetically appealing images at different energy levels of Figure 1 are used as reference images, and the distances between the energies of the tested image should be as close as possible to the distances of the reference image R_i_, as described by (15); furthermore, the equation described by (14) should be also satisfied. In other words, M should be maximized and Md should be minimized

(15) $$\mathrm{Md}=\left|\underset{i}{\sum }\mathrm{Distance}\left(\mathrm{Ri}\right)\;-\;\underset{i}{\sum }\mathrm{Distance}\left(\mathrm{Li}\right)\right|$$

where Distance(R_i_) is the distance between the energy of the ith level and the energy of the i + 1 level, and the energy of the first level only should be the same. The metrics will be calculated on the center part of the image, since it gets most of the attention, where 20 pixels from each side of the image will be neglected. Figure 8 shows the combination of the two metrics where the sum of the entropies and the energies of the three levels is shown after scaling each energy and entropy to value between 0 and 1.

## 4. Results

**Figure 9:**
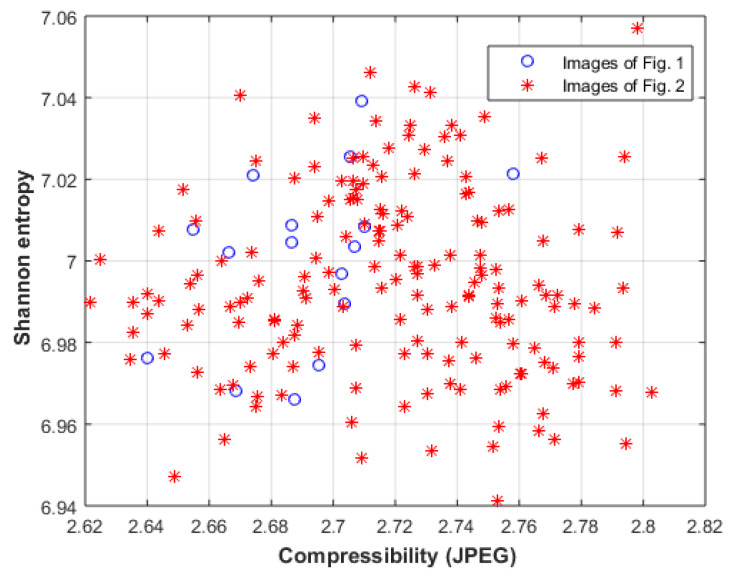
Shannon entropy vs image compressibility for images of Figure 1 and Figure 2.

Due to the small number of images in the two datasets, the proposed approach cannot be compared to deep-learning-based approaches [47,48,49,50,51]. The proposed approach will be compared with three related approaches; the first one is based on the Birkhoff model [52,53], where Shannon entropy and image compressibility are used to represent the order and complexity of the Birkhoff model. Figure 9 shows the Shannon entropy and image compressibility (the ratio between the original and the compressed image using the JPEG method) for the images of Figure 1 and Figure 2. The results show that the two groups of images cannot easily be classified using this approach.

**Figure 10:**
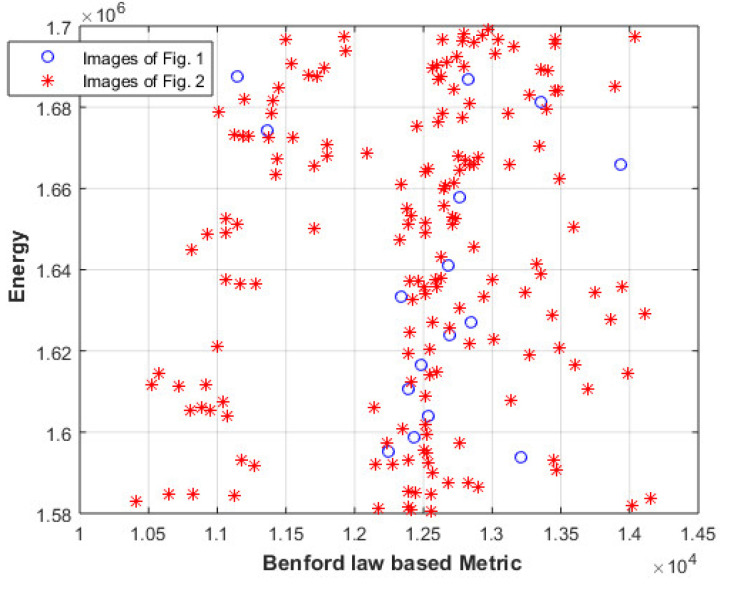
The difference between the histograms of the images of Figure 1 and Figure 2, and the histogram described by Benford law.

Then, the proposed approach will be compared with an approach based on Benford law [54], where the histogram of the image is compared with the histogram described by Benford law. Figure 10 shows the difference between the histograms of the images of Figure 1 and Figure 2, and the histogram described by Benford law. The results also show that the two groups of images cannot be easily classified using this approach.

**Figure 11:**
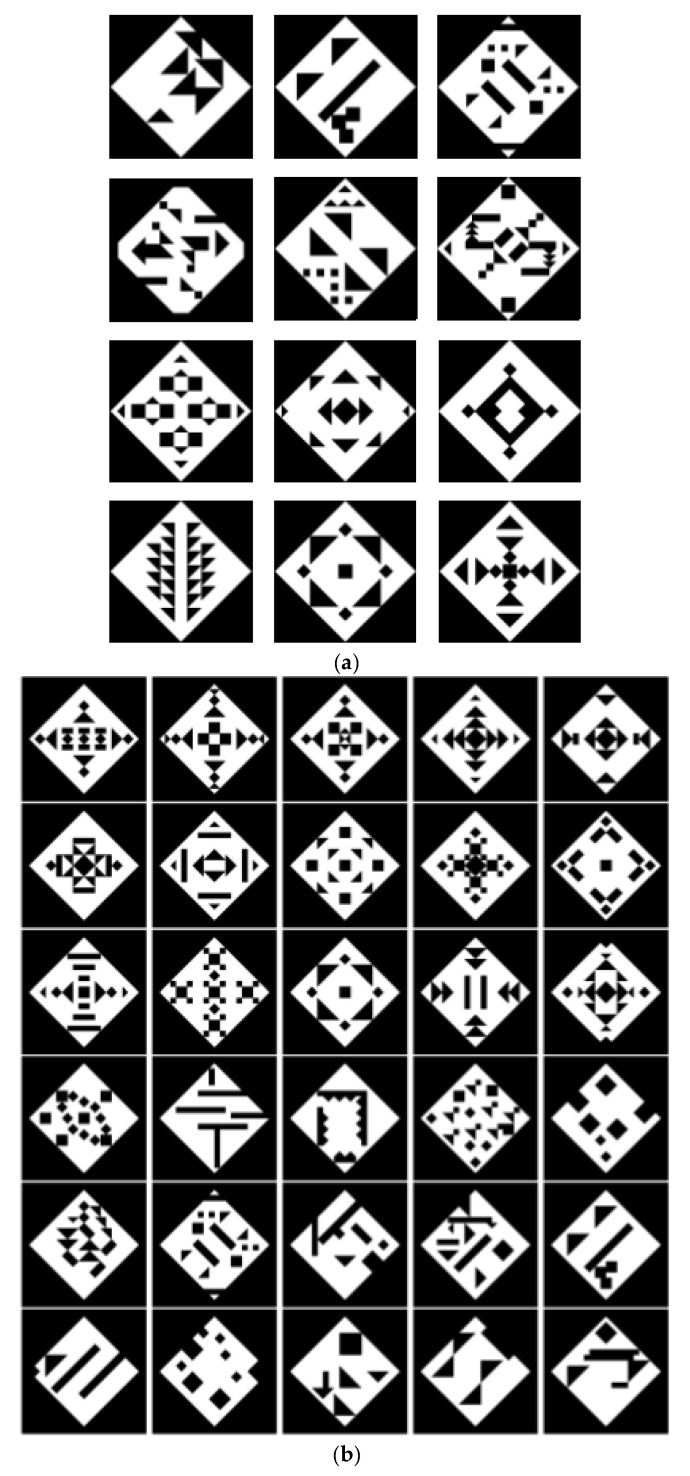
Patterns from the dataset proposed in [39], (**a**) ordered from not beautiful (left) to beautiful (right) line by line. (**b**) Ordered from beautiful (left) to not beautiful (right) line by line.

**Figure 12:**
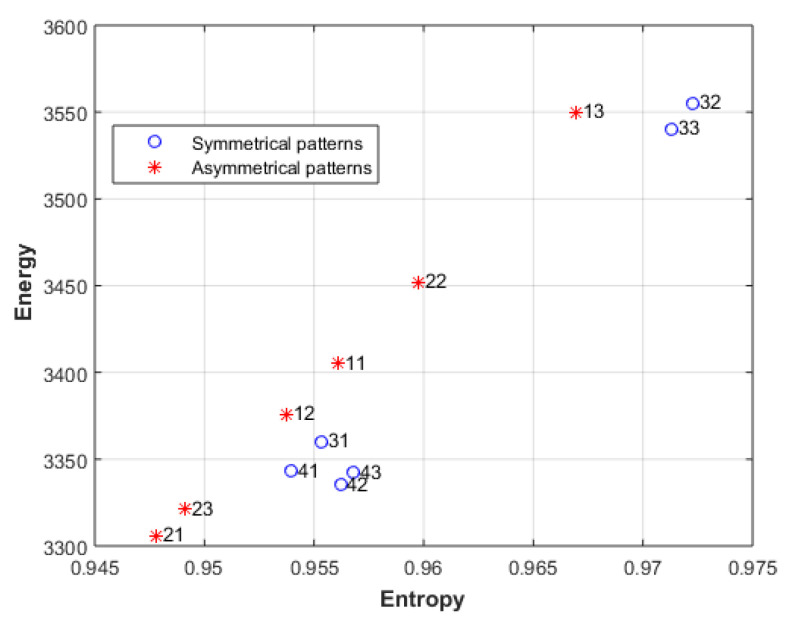
The energy and the entropy of the first level of the images in Figure 11a.

**Figure 13:**
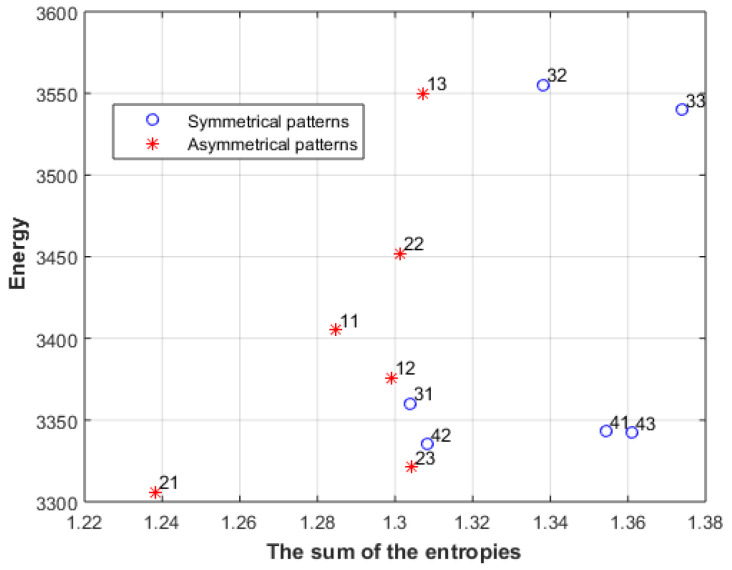
The sum of the entropies of the first two levels of the images in Figure 11a.

**Figure 14:**
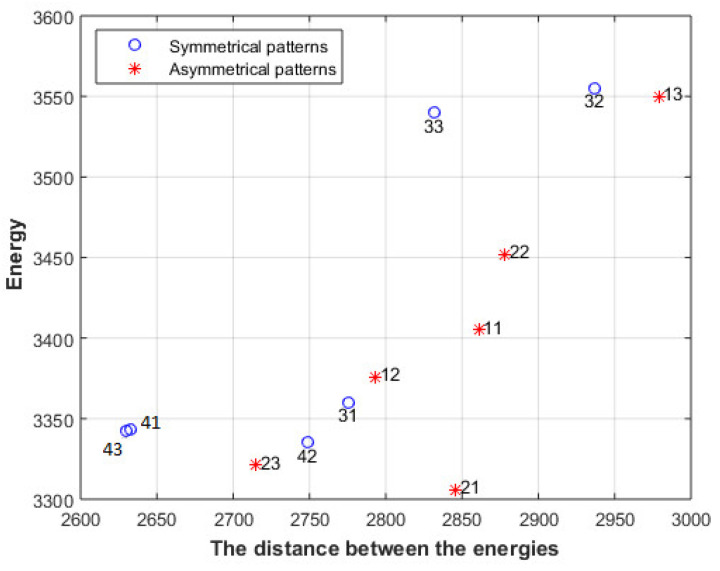
The distance between the energies of the first two levels of the images in Figure 11a.

**Figure 15:**
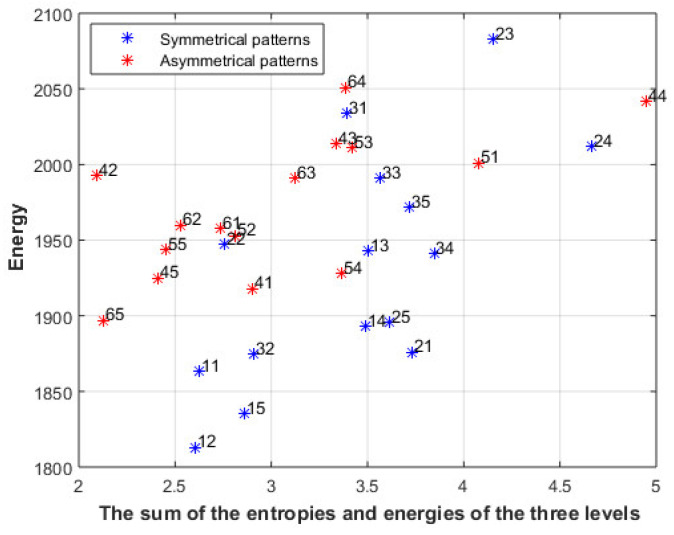
The sum of the entropies and the energies of the three levels of the images in Figure 11b.

To further test the proposed approach, we will test it on the dataset proposed in [39]. Figure 11 shows the patterns of the set. In Figure 11a, the first two lines represent asymmetrical patterns and the last two lines represent symmetrical patterns. Fifty-five persons rated the patterns; the patterns start from not beautiful (left), and move to beautiful (right) line by line. In Figure 11b, the first three lines represent symmetrical patterns and the last three lines represent asymmetrical patterns, ordered in lines from the most beautiful pattern starting in the upper left corner to the least beautiful pattern. The number next to each pattern in Figure 12, Figure 13, Figure 14 and Figure 15 represents the line number and the position of the pattern in the line (starting from left to right). For instance, 43 is the third pattern in line four. 

Figure 12 shows the energy and the entropy of the first level; the results show that the symmetrical patterns of line 3 and line 4 have higher entropy than the asymmetrical patterns when the same energy is used. This matches with the rating given by the fifty-five persons and with several studies [55,56,57,58], which showed consistent preferences for symmetry. The patterns 41, 42, and 43 have roughly the same energy, but the entropy of 43 is larger than the entropy of 42, which is larger than the entropy of 41.

Figure 13 shows the sum of the entropies of the first two levels after converting all levels to black and white images; again, the symmetrical patterns of line 3 and line 4 have a higher sum than the other patterns when the same energy is used. For instance, patterns 13, 32, and 33 have roughly the same energy, but the sum of 33 is larger than the sum of 32, which is larger than the sum of 13. This also matches with the rating of the fifty-five persons. We can also see that the patterns 11 and 21 have lower sum than the other patterns. 

Figure 14 shows the distance between the energies of the first two levels. The symmetrical patterns of line 3 and line 4 have a lower distance than the other patterns when the same energy is used. For instance, the patterns 13, 32, and 33 have roughly the same energy, but the distance of 33 is lower than the distance of 32, which is lower than the distance of 13. The patterns 41, 42, and 43 also have roughly the same energy, but the distance of 43 is lower than the distance of 42; however, 42 has higher distance than 41. We can also see that the patterns 11 and 21 have higher distance than the other patterns. These results show a close match with the rating given by the fifty-five persons.

Figure 15 shows the results using the images in Figure 11b; again, the symmetrical patterns of the first three lines show a higher sum than other asymmetrical patterns when the same energy is used; however, there are some differences between the sum and the ranking of the users within these two groups.

**Figure 16:**
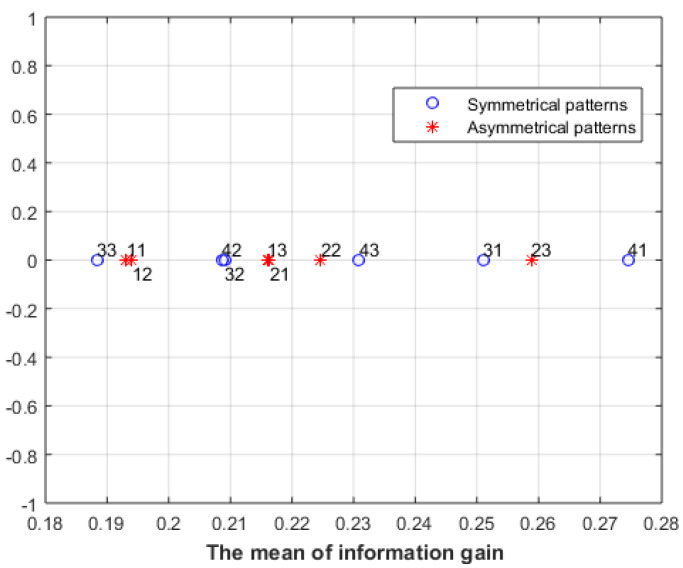
The mean information gain of the images in Figure 11a.

Figure 16 shows the results of applying an information-gain-based approach proposed in [29] for the images in Figure 11a. The results show a link between information gain and empirical aesthetic judgement in the case of the asymmetrical patterns, but not for the symmetrical patterns. We can see that the ordering of the information gain for the asymmetrical patterns agrees with the users’ rating. However, this is not the case for the symmetrical patterns.

**Figure 17:**
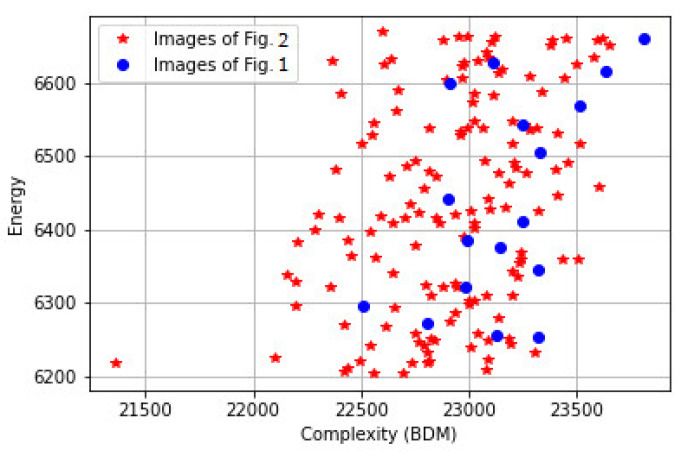
The energy and complexity (BDM) of the images in Figure 1 and Figure 2.

**Figure 18:**
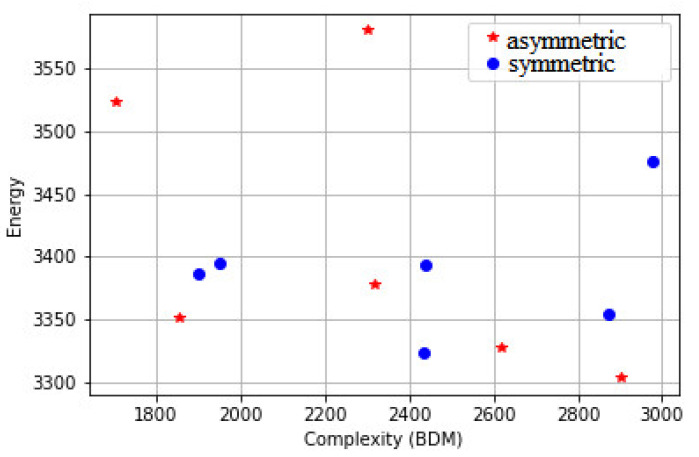
The energy and complexity (BDM) of the images in Figure 11a.

Figure 17 and Figure 18 show the algorithmic complexity as approximated by the Block Decomposition Method (BDM) [59,60] after converting the patterns to black and white, since the method does not yet support a large number of values. The method performs better on the first dataset than the second dataset, where we can see that aesthetically appealing patterns tend to have a higher complexity. However, it is still clear that this method is less accurate in comparison with the proposed approach.

## 5. Discussion

The results show that the proposed model is more accurate at classifying aesthetically appealing visual patterns. The results suggest that aesthetically appealing patterns of the two datasets are more likely to deliver a higher amount of information in comparison with less aesthetically appealing patterns when the same amount of energy is used. The results also suggest that the distances between the energies of the levels are more likely to be different for aesthetically appealing patterns. One limitation of the proposed approach is that few aesthetically appealing patterns show a lower M value and higher Md value than the less aesthetically appealing patterns, as can be seen in Figure 8. Future work will improve the proposed model to increase the classification accuracy.

To give a more intuitive analysis of the results, we will take two extreme cases: the first one is an image with only one color, and the second one is an image with equal probabilities for all colors. The first case will produce a distribution of one pulse at one energy level, while the second case will produce a flat distribution. In the case of music, the first case will give a piece with only one note repeated many times, and the second case will produce a piece with all possible notes; in both cases, no aesthetically appealing patterns will be produced, as the first pattern will be too regular and the second one will be too random. The aesthetically appealing patterns represent a balance between these two extreme cases, and the closer we get to the Maxwell–Boltzmann distribution, the higher the aesthetic score of the pattern. Now, if we take one aesthetically appealing pattern and rearrange the pixels randomly, we will obtain a random pattern that has the same distribution; however, the gradient (which decodes the spatial distribution of pixels) of this random pattern will produce a distribution closer to the flat distribution than the gradient of the original pattern. Similarly, if we arrange the aesthetically appealing pattern such that the pixels with the same values are close to each other, the gradient of the resulted pattern will produce a distribution closer to a pulse than the gradient of the original pattern, and again the distribution of the gradient of aesthetically appealing patterns represents a balance between these two extreme cases, and the closer we get to the Maxwell–Boltzmann distribution, the higher the aesthetic score of the pattern.

The proposed approach agrees with the intuition of many scientists, who link the concept of beauty with the ability to cover the largest possible number of empirical facts using the smallest possible number of axioms or hypotheses [61,62]. Similarly, the proposed approach suggests that aesthetically appealing patterns should deliver the largest possible amount of information using the same amount of energy. The relation between aesthetically appealing patterns and the balance between randomness and regularity was also suggested by many researchers. The proposed approach uses a statistical mechanics formulation that links the energy and the entropy, which was a crucial link to constrain the complexity of the pattern by the energy, and hence achieve a balance between randomness and regularity.

The proposed approach has shown an interesting link between information theory and the aesthetic rating of the users of the two datasets. The meaning and the deeper relation of the link between information theory and aesthetic are to be further investigated in future work. Finding the most fundamental law or the optimization process that underlies aesthetically appealing patterns would be of great interest for the research in this area and for many applications [63,64,65,66,67,68,69,70,71,72,73,74,75,76,77,78,79]. It is interesting to see whether the proposed approach has any link to the aesthetic judgment mechanism in the brain, and how is that related to information theory. Pursuing these research directions holds a great promise for a deeper understanding of many important phenomena.

## 6. Conclusions

A novel approach to classify aesthetically appealing images was presented in this paper. The proposed approach showed that aesthetically appealing images of the two datasets are more likely to deliver a higher amount of information over multiple levels in comparison with less aesthetically appealing images when the same amount of energy is used. The results have shown that the proposed approach was more accurate in classifying aesthetically appealing patterns. Future work will try to apply this approach to other types of images.

## Data Availability

Not applicable.
